# Editorial on, “Amyloid-beta clearance in Alzheimer's disease”

**DOI:** 10.3389/fnagi.2014.00310

**Published:** 2014-11-20

**Authors:** Robert A. Marr

**Affiliations:** Department of Neuroscience, Rosalind Franklin University of Medicine and Science/Chicago Medical SchoolNorth Chicago, IL, USA

**Keywords:** Alzheimer's disease, amyloid-beta, clearance, proteases, LDL receptors, signal transduction

Alzheimer's disease (AD) is the most common form of dementia and is exacting a tremendous economic and personal toll on nations as well as families throughout the world and this toll will continue to grow over the passing decades. This crisis is compounded by the lack of effective treatments for this disease and the widespread failure of a multitude of therapeutic clinical trials targeting many identified disease pathways including amyloid-beta (Aβ), tau, inflammation, oxidative stress and more (reviewed in Schneider et al., 2014). Regardless of these failures, all of these disease pathways are likely key players in disease pathogenesis and improved therapeutics as well as better clinical study design may yet prove effective. Because reduced clearance of Aβ has been clinically linked to AD (Mawuenyega et al., 2010), it is likely important to disease pathogenesis. This research topic focuses on aspects of Aβ clearance in AD and its therapeutic relevance.

The research topic on Aβ clearance in AD in Frontiers in Aging Neuroscience has produced a highly informative collection of reviews, mini-reviews, opinions, hypothesis and theory articles, as well as original research articles that cover key aspects related to Aβ clearance. These aspects include proteolytic degradation, low-density lipoprotein (LDL) receptor related clearance, cellular signaling pathways related to clearance, and transport/phagocytosis.

With regards to proteases, Marr and Hafez produced a mini-review that provides a concise rationale for the amyloid hypothesis of AD and updates recent finding related to the neprilysin-2 (NEP2) enzyme and its potential role in AD (Marr and Hafez, 2014). The other articles concerning proteases present new angles of looking at this topic. Malcolm Leissring contributed an opinion specifically on the critical role of clearance of intracellular Aβ in disease pathogenesis; while Miners et al. gave us a review focused on a dual role for these enzymes in both Aβ clearance and cerebral perfusion as well as how these process may interact (Leissring, 2014; Miners et al., 2014). Nalivaeva et al. produced a comprehensive review of key Aβ-degrading enzymes focusing on the epigenetic regulation of their expression. This review also discusses transthyretin (TTR), an Aβ binding protein involved in its transport/clearance (Nalivaeva et al., 2014). Related to this, the original article contributed by Philibert et al. includes the characterization of TTR binding to Aβ and its ability to prevent oligomerization. This original article also characterizes the direct Aβ degrading activity of thimet oligopeptidase (EC 3.4.24.15) (Philibert et al., 2014).

On the subtopic of LDL receptors, Kanekiyo and Bu provided a review on the role of LDL receptor-related protein 1 (LRP1) in Aβ endocytosis and signal transduction in relation to AD pathogenesis (Kanekiyo and Bu, 2014). Also regarding endocytosis (and LDL receptors), Zhao et al. contributed an original article on the effects of aging and apolipoprotein E status on glial uptake of lentiviral-expressed Aβ, showing that apoE4 results in less efficient microglial clearance of Aβ (Zhao et al., 2014). Following on the subtopic of glial uptake, Bhattacharjee et al. produced an opinion focusing on miRNA-34a as a regulator of the phagocytosis sensor-receptor, TREM2, and its potential role in AD (Bhattacharjee et al., 2014). Zolezzi et al. provided a comprehensive review of AD pathogenesis and Aβ regulation focusing on the PPAR signaling pathway; while Hernandez-Rapp et al. wrote a mini review on PrP^c^ signaling and its effects on Aβ clearance (Hernandez-Rapp et al., 2014; Zolezzi et al., 2014).

These articles also discuss the therapeutic implications of their topics. Related to this, Spencer and Masliah provided a review on the state of immunotherapy for AD and future perspectives on this type of Aβ clearance-related therapeutic approach (Spencer and Masliah, 2014). Finally, Qiu and Zhu produced a hypothesis and theory article on the relevance of amylin to AD therapy (Qiu and Zhu, 2014). Therefore, this compilation of articles is highly relevant to the study and development of AD therapies.

## Conflict of interest statement

The author declares that the research was conducted in the absence of any commercial or financial relationships that could be construed as a potential conflict of interest.
